# Adaptive dynamic programming for coordinated control of a dual-arm reconfigurable manipulator with unknown object constraints

**DOI:** 10.3389/frobt.2026.1923646

**Published:** 2026-09-03

**Authors:** Zebin Ji, Yi Qin, Qiang Pan, Hucheng Jiang, Bing Ma

**Affiliations:** 1 Department of Control Science and Engineering, Changchun University of Technology, Changchun, China; 2 Aviation University of Air Force, Changchun, China

**Keywords:** adaptive dynamic programming, dual-arm coordinated control, neural network, optimal control, reconfigurable manipulator

## Abstract

Concentrating on various types of grasped objects, this work presents a distributed coordinated control scheme for a dual-arm reconfigurable manipulator (DRM) via adaptive dynamic programming (ADP). Kinematic and force analyses are performed to formulate the dynamics of a single-arm manipulator and the object using joint torque feedback technology and the Newton–Euler formulation. To address uncertainties associated with the grasped object, a gradient model-based adaptive algorithm is developed for online estimation of the grasp matrix relating the object to the DRM. By employing an adaptive observer to identify unknown dynamic terms, an enhanced optimal performance index is constructed that incorporates both object tracking performance and internal force effects within the manipulator. The performance index function is approximated by only a critic neural network, and the optimal control policy is obtained by policy iteration. Lyapunov stability analysis demonstrates that internal force error, position tracking error, and critic network weight approximation error are all uniformly ultimately bounded. Experimental results confirm the effectiveness of the developed coordinated control approach.

## 1 Introduction

A dual-arm manipulator demonstrates formidable capabilities in collaborative work and multitasking, exhibiting exceptional coordination and flexibility (Cao et al., 2024). A dual-arm manipulator showcases promising application prospects across diverse fields, such as industrial automation, minimally invasive surgery, precise harvesting of fruits and vegetables, household management, object transportation, and multi-object assembly. To accomplish these tasks, enhancing the flexibility, autonomy, and versatility of the dual-arm manipulator is essential. A conventional dual-arm manipulator, however, is typically designed with a fixed configuration to execute specific tasks, often requiring designers to manually derive and formulate a system model based on a predetermined structure. Due to the modular design, a reconfigurable manipulator (An et al., 2025b; Ji et al., 2025) can be assembled in various configurations. By partially replacing components and reconfiguring the system, a reconfigurable manipulator can effectively enhance fault tolerance while increasing overall flexibility and versatility. Consequently, the dual-arm reconfigurable manipulator system holds significant research value.

During object transportation, the dual-arm manipulator often forms a closed-chain constraint, which significantly enhances precision and stability but may also lead to mutual interference. Consequently, coordinated manipulation in the dual-arm and the increased degrees of freedom of the manipulator itself compound system complexity, thereby increasing the demand for coordinated control. The traditional coordinated control method relies heavily on a precise system dynamic model, computationally intensive kinematic calculations, and extensive offline training, which limits applicability in real-time scenarios and adaptability to dynamic tasks. For dual-arm transportation tasks, adjusting traditional impedance control parameters proves particularly challenging due to environmental uncertainties. Although impedance control (Song et al., 2025) can achieve interactive tasks required by an end-effector and object, and it can be applied without any switching operation between contact and non-contact conditions, its precision in controlling both the position of the grasped object and the contact force is lower than hybrid force/position control. It may limit impedance control applicability in certain scenarios, such as surgery, manufacturing, precision welding, and similar fields. Hybrid force/position control (Zhao et al., 2022; Sardarmehni and Song, 2023) enables simultaneous coordination of force and displacement in different directions, thereby finding broad application in dual-arm coordinated control. Additionally, when a dual-arm manipulator system grasps tool of varying sizes, the geometric uncertainty can alter the grasp matrix. This, in turn, affects the closed-loop kinematics of the interconnected system, making it difficult to achieve measurable decoupling of grasping force, let alone internal force tracking. Therefore, estimation of the tool becomes necessary.

To enhance the control performance of the manipulator system while reducing the energy consumption required by the controller, optimal control (Sun and Yuan, 2026; Luo et al., 2025) for a manipulator is of comparable significance. As a crucial component of modern control theory, optimal control focuses on the fundamental problem of selecting the control policy that optimizes certain performance metrics of a given controlled system. For the linear quadratic regulator problem, which involves a linear system as the controlled plant and a quadratic cost function, an optimal control strategy is primarily derived by addressing the Riccati equation. The approach has now become a mature and well-established component of optimal control theory. However, for the numerous nonlinear systems prevalent in practical engineering applications, obtaining the optimal control strategy requires addressing the Hamilton–Jacobi–Bellman (HJB) equation that constitutes a class of nonlinear partial differential (or difference) equations, making it difficult to derive analytical optimal solution.

Adaptive dynamic programming (ADP) (An et al., 2023; Ting and Aiguo, 2019; Yuan et al., 2025) serves as an efficient approach for addressing optimal control problems in nonlinear systems. In an ADP framework, neural networks are designed to approximate the performance index function and estimate a solution to the HJB equation, thereby providing an effective strategy for tackling the analytical challenges associated with nonlinear optimal control. Due to its strong advantages in solving nonlinear optimal control problems, ADP has attracted widespread attention from scholars in recent years. ADP has yielded substantial results in addressing challenges such as discrete-time (Sun and Xu, 2024; Wei et al., 2019; Luo et al., 2026), continuous-time (Sun et al., 2023; Hyatt et al., 2020; An et al., 2025a) data-driven (Liu et al., 2026; Zhang et al., 2022; Zhang and Liu, 2025), event-triggered (Diao et al., 2021; Qin et al., 2026; Conlon et al., 2024), vehicle platoon (Wang et al., 2026), and interconnected (Xia et al., 2025) optimal control for complex nonlinear systems.

The contributions of this study are mainly embodied in two dimensions:

This article addresses the scenario of a dual-arm reconfigurable manipulator system grasping an unknown object by designing an optimal force/position controller that enhances control accuracy while reducing energy consumption. Through estimation of the system’s grasp matrix, decoupling of grasping force is achieved, thereby enabling internal force tracking.

The adaptive dynamic programming method is introduced into force/position control. Based on the designed fusion error function, the approach simultaneously optimizes position tracking error and internal force error. In addition, only the critic network is used without slowing the response of the system or accumulating error. The proposed control method is further implemented and validated on the actual experimental platform.

## 2 System description and preliminaries

### 2.1 Kinematic analysis

Denote $x_{o}\in R^{p}$ as the coordinate vector of the object’s center of mass (COM). Denote $x_{ei}\in R^{p\times 1}$ as the end-effector position of the i th reconfigurable manipulator. The relationship between $x_{o}$ and $x_{ei}$ can be described as in Equation 1

$${\dot{x}}_{ei}=J_{oi}{\dot{x}}_{o},$$ (1)

where $J_{oi}\left(x_{o}\right)$ represents the grasping matrix. It can be obtained by $J_{oi}\left(x_{o}\right)=\left[\begin{array}{cc} I_{3} & 0_{3}\\ S_{i} & I_{3} \end{array}\right]$ for i=1,2 . $S_{i}=\left[\begin{array}{ccc} 0 & -S_{iz} & S_{iy}\\ S_{iz} & 0 & -S_{ix}\\ -S_{iy} & S_{ix} & 0 \end{array}\right]$ , $r_{\mathrm{ori}}={\left[S_{ix},S_{iy},S_{iz}\right]}^{T}$ is vector from the COM of the object being grasped under the world coordinate system to the end of the manipulator. The relationship between $x_{ei}$ and $q_{i}$ can be described as in Equation 2:

$${\dot{x}}_{ei}=J_{si}{\dot{q}}_{i},$$ (2)

where $q_{i}\in R^{n_{i}\times 1}$ denotes the joint position of the reconfigurable manipulator, ${\dot{q}}_{i}\in R^{n_{i}\times 1}$ is the velocity of i th reconfigurable manipulator, $n_{i}$ is the degrees of freedom (DOF) of the manipulator, and $J_{si}\in R^{p\times n_{i}}$ is the Jacobian matrix from the end-effector to $q_{i}$ .

The forward kinematic of i th reconfigurable manipulator is

$$x_{o}=\phi_{i}\left(q_{i}\right),\;i=1,2.$$ (3)

Under the assumption that the point of force application between the grasped object and the end-effector coincides with the origin of the kinematic constraint coordinate frame and neglecting the volume of the end-effector, the partial derivative of Equation 3 can be expressed as follows:

$${\dot{x}}_{o}={\dot{\phi }}_{i}\left(q_{i}\right)=J_{\phi i}\left(q_{i}\right){\dot{q}}_{i},$$ (4)

$${\ddot{x}}_{o}={\dot{J}}_{\phi i}\left(q_{i}\right){\dot{q}}_{i}+J_{\phi i}\left(q_{i}\right){\ddot{q}}_{i},$$ (5)

where $J_{\phi i}\left(q_{i}\right)\in R^{p\times n_{i}}$ is the Jacobian matrix from $q_{i}$ to the COM of the grasped object, ${\ddot{q}}_{i}\in R^{n_{i}\times 1}$ denotes the joint acceleration of the i th reconfigurable manipulator, and the Jacobian matrix $J_{\phi i}\left(q_{i}\right)$ and the grasp matrix are in Equation 6

$$J_{\phi i}\left(q_{i}\right)=J_{oi}{\left(x_{o}\right)}^{+}J_{si}\left(q_{i}\right).$$ (6)

### 2.2 Force analysis of a dual-arm reconfigurable manipulator system

Let $F_{o}\in R^{p}$ represent the resultant force that the manipulators exert on the COM of the grasped object. This force can be expressed as in Equation 7

$$F_{o}=-\overset{2}{\underset{i=1}{\sum }}F_{\mathrm{cei}},$$ (7)

where $F_{\mathrm{cei}}\in R^{p}$ is force acting on centroid of grasped object by i th reconfigurable manipulator. The relationship between end-effector force $F_{ei}$ and $F_{\mathrm{cei}}$ is in Equation 8

$$F_{\mathrm{cei}}=J_{oi}^{\text{T}}F_{ei}.$$ (8)

$F_{ei}$ consists of the external force used to drive the object’s motion and the internal force for maintaining the object’s stability during grasping as in Equation 9:

$$F_{ei}=F_{Ji}+F_{Ii},$$ (9)

where $F_{Ji}={\left(J_{oi}^{\text{T}}\left(x_{o}\right)\right)}^{+}F_{o},F_{Ii}=IF_{ei}$ , and $I=I-{\left(J_{oi}^{\text{T}}\left(x_{o}\right)\right)}^{+}J_{oi}^{\text{T}}\left(x_{o}\right)$ denotes the zero space matrix of $J_{oi}^{\text{T}}\left(x_{o}\right)$ . According to the principle of load distribution, the relationship between the combined force $F_{o}$ and component $F_{oi}$ of the object is expressed as in Equation 10:

$$F_{oi}=\frac{1}{2}F_{o}.$$ (10)

### 2.3 Dynamic analysis

Combining load distribution and force analysis, the dynamic of the i th reconfigurable manipulator is as follows:

$$I_{mi}{\gamma_{i}}^{2}{\ddot{q}}_{i}+\gamma_{i}f_{i}\left(q_{i},{\dot{q}}_{i}\right)+\tau_{Si}=\gamma_{i}\tau_{i}-J_{si}^{\text{T}}\left[\frac{1}{2}{\left(J_{oi}^{\text{T}}\right)}^{+}F_{o}+F_{Ii}\right],$$ (11)

where $I_{mi}\in R^{n_{i}\times n_{i}}$ indicates the rotational inertia of the rotor, $\gamma_{i}\in R^{n_{i}\times n_{i}}$ denotes the gear ratio, $f_{i}\left(q_{i},{\dot{q}}_{i}\right)\in R^{n_{i}}$ is the joint friction torque, $\tau_{Si}\in R^{n_{i}}$ represents the coupling torque, and $\tau_{i}\in R^{n_{i}}$ is the output torque of the DC motor.

The following equation describes the model of the object in the workspace:

$$M_{o}\left(x_{o}\right){\ddot{x}}_{o}+C_{o}\left(x_{o},{\dot{x}}_{o}\right){\dot{x}}_{o}+g_{o}\left(x_{o}\right)=F_{o},$$ (12)

where $M_{o}\left(x_{o}\right)\in R^{p\times p}$ is the positive definite symmetry inertia matrix, $C_{o}\left(x_{o},{\dot{x}}_{o}\right)\in R^{p\times p}$ is the centrifugal torque matrix and the Coriolis torque matrix of the object, and $g_{o}\left(x_{o}\right)\in R^{p}$ is the gravitational term of the object.

Given the tool reference model parameters $M_{D}$ , $C_{D}$ , and $g_{D}$ , Equation 12 can be rewritten as follows:

$$\begin{array}{cc} F_{o} & =\left(M_{D}\left(x_{o}\right)+\tilde{M}\left(x_{o}\right)\right){\ddot{x}}_{o}+\left(C_{D}\left(x_{o},{\dot{x}}_{o}\right)+\tilde{C}\left(x_{o},{\dot{x}}_{o}\right)\right){\dot{x}}_{o}+\left(g_{D}\left(x_{o}\right)+\tilde{g}\left(x_{o}\right)\right)\\ & =M_{D}\left(x_{o}\right){\ddot{x}}_{o}+C_{D}\left(x_{o},{\dot{x}}_{o}\right){\dot{x}}_{o}+g_{D}\left(x_{o}\right)+e_{Mi}, \end{array}$$ (13)

where $\begin{array}{cc} & e_{Mi}=\tilde{M}\left(x_{o}\right){\ddot{x}}_{o}+\tilde{C}\left(x_{o},{\dot{x}}_{o}\right){\dot{x}}_{o}+\tilde{g}\left(x_{o}\right)\\ & =\tilde{M}\left(x_{o}\right)J_{\phi i}{\ddot{q}}_{i}+\left(\tilde{M}\left(x_{o}\right){\dot{J}}_{\phi i}+\tilde{C}\left(x_{o},{\dot{x}}_{o}\right)J_{\phi i}\right){\dot{q}}_{i}+\tilde{g}\left(x_{o}\right) \end{array}$ is the reference model error. $\tilde{M}\left(x_{o}\right)=M_{o}\left(x_{o}\right)-M_{D}\left(x_{o}\right),\tilde{C}\left(x_{o},{\dot{x}}_{o}\right)=C_{o}\left(x_{o},{\dot{x}}_{o}\right)-C_{D}\left(x_{o},{\dot{x}}_{o}\right),\tilde{g}\left(x_{o}\right)=g_{o}\left(x_{o}\right)-g_{D}\left(x_{o}\right)$ is the error term.

Combining Equations 11, 13, kinematic Equation 4, and Equation 5, the dynamic equation of a single manipulator with an object can be expressed as follows:

$$\begin{array}{cc} u_{i} & =\left[I_{mi}{\gamma_{i}}^{2}+\Gamma M_{D}\left(x_{o}\right)J_{\phi i}\right]{\ddot{q}}_{i}+\left[\Gamma M_{D}\left(x_{o}\right){\dot{J}}_{\phi i}+\Gamma C_{D}\left(x_{o},{\dot{x}}_{o}\right)J_{\phi i}\right]{\dot{q}}_{i}\\ & \;+F_{i}+\tau_{Si}+\tau_{i}+\Gamma g_{D}\left(x_{o}\right)+\Gamma e_{Mi}, \end{array}$$ (14)

where $\Gamma =\frac{1}{2}J_{si}^{\text{T}}{\left(J_{oi}^{\text{T}}\right)}^{+}$ , $\tau_{i}=J_{si}^{\text{T}}F_{Ii}$ , $F_{i}=\gamma_{i}f_{i}\left(q_{i},{\dot{q}}_{i}\right)$ and $u_{i}=\gamma_{i}\tau_{i}$ .

Based on Equation 14, the state space equation of the dynamic for a dual-arm reconfigurable manipulator is as in Equation 15:

$$\left\{\begin{array}{cc} & {\dot{x}}_{1i}=x_{2i}\\ & {\dot{x}}_{2i}={▵}_{Di}+N_{Di}f_{i}+N_{Di}\left(u_{i}-\tau_{Si}-\tau_{i}\right), \end{array}\right.$$ (15)

where $x_{i}={\left[x_{1},x_{2}\right]}^{\text{T}}={\left[q_{i},{\dot{q}}_{i}\right]}^{\text{T}}$ is the state vector.

$${▵}_{Di}=-{\left[I_{mi}{\gamma_{i}}^{2}+\Gamma_{i}M_{D}\left(x_{o}\right)J_{\phi i}\right]}^{-1}\,\left[\left(\Gamma_{i}\,M_{D}\,\left(x_{o}\right)\,{\dot{J}}_{\phi i}+\Gamma_{i}\,C_{D}\,\left(x_{o},{\dot{x}}_{o}\right)\,J_{\phi i}\right)\,{\dot{q}}_{i}+\Gamma_{i}\,g_{D}\,\left(x_{o}\right).\right]$$

$N_{Di}={\left[I_{mi}{\gamma_{i}}^{2}+\Gamma_{i}M_{D}\left(x_{o}\right)J_{\phi i}\right]}^{-1}$ , and $f_{i}=-F_{i}-\Gamma_{i}e_{Mi}$ is an unknown term.

### 2.4 Estimation of the object’s COM

First, assume that the initial position of the target object’s COM is random and calculate the estimated grasping matrix ${\hat{J}}_{oi}$ based on this initial value. The estimated acceleration ${\hat{\ddot{X}}}_{o}$ of the target object’s COM is obtained from the COM dynamic equation of the target object as follows:

$$M_{o}\left(x_{o}\right){\hat{\ddot{x}}}_{o}+g_{o}\left(x_{o}\right)={\hat{F}}_{o},$$ (16)

where ${\hat{F}}_{o}$ is the estimated resultant force on the COM of the target object.

According to Equation 16, the estimated acceleration ${\hat{\ddot{x}}}_{o}$ of the COM and the component vector ${{\hat{F}}_{o}}^{{}^{\circ}}$ of the resultant force ${\hat{F}}_{o}$ acting on COM of the target tool satisfy the following relationship.

$$m_{o}{\hat{\ddot{x}}}_{o}={{\hat{F}}_{o}}^{{}^{\circ}}-g_{o}\left(x_{o}\right),$$ (17)

$${{\hat{F}}_{o}}^{{}^{\circ}}=F_{e1}+F_{e2},$$ (18)

where $m_{o}$ is the weight of the target tool; $F_{e1}$ and $F_{e2}$ represent the contact forces exerted by the left and right arms of the dual-arm reconfigurable manipulator (DRM) on the target object, respectively.

Combining Equation 17 and Equation 18, we obtain in Equation 19

$$m_{o}{\hat{\ddot{x}}}_{o}=F_{e1}+F_{e2}-g_{o}\left(x_{o}\right).$$ (19)

Therefore, the estimated acceleration ${\hat{\ddot{x}}}_{o}$ of object’s COM can be expressed as in Equation 20:

$${\hat{\ddot{x}}}_{o}=m_{o}^{-1}\left(F_{e1}+F_{e2}-g_{o}\left(x_{o}\right)\right).$$ (20)

Define the estimated target object’s COM velocity ${\hat{\dot{x}}}_{o}\left(k\right)$ and the estimated target object’s COM position ${\hat{x}}_{o}\left(k\right)$ as in Equation 21

$${\hat{\dot{x}}}_{o}\left(k\right)={\hat{\ddot{x}}}_{o}\left(k-1\right)\Delta t+{\hat{\dot{x}}}_{o}\left(k-1\right),$$ (21)

$${\hat{x}}_{o}\left(k\right)={\hat{\dot{x}}}_{o}\left(k-1\right)\Delta t+{\hat{x}}_{o}\left(k-1\right).$$ (22)

Through the given kinematic relationship, the estimated values of the two contact points between the two end-effectors of the dual-arm reconfigurable manipulator and the target object are obtained:

$$\left\{\begin{array}{cc} & {\hat{x}}_{c1}\left(k\right)={\hat{x}}_{o}\left(k\right)+R_{o}{\hat{r}}_{\mathrm{or1}}\\ & {\hat{x}}_{c2}\left(k\right)={\hat{x}}_{o}\left(k\right)+R_{o}{\hat{r}}_{\mathrm{or2}}, \end{array}\right.$$ (23)

where $R_{o}$ is the transformation matrix from the target object coordinate system to the world coordinate; ${\hat{r}}_{\mathrm{ori}}={\left[{\hat{S}}_{ix},{\hat{S}}_{iy},{\hat{S}}_{iz}\right]}^{\text{T}}$ is the estimated value of the direction vector of the end-effector from the COM of the target object to the end of the i th arm in the world coordinate.

The estimated error in defining the position of the two contact points is defined as follows:

$$\left\{\begin{array}{cc} & e_{1}=x_{c1}-{\hat{x}}_{c1}\\ & e_{2}=x_{c2}-{\hat{x}}_{c2} \end{array}\right.$$ (24)

Combining Equations 22-24, we obtain as Equation 25

$$\left\{\begin{array}{cc} & e_{1}=x_{c1}-\left({\hat{\dot{x}}}_{o}\left(k-1\right)\Delta t+{\hat{x}}_{o}\left(k-1\right)+R_{o}{\hat{r}}_{\mathrm{or1}}\right)\\ & e_{2}=x_{c2}-\left({\hat{\dot{x}}}_{o}\left(k-1\right)\Delta t+{\hat{x}}_{o}\left(k-1\right)+R_{o}{\hat{r}}_{\mathrm{or2}}\right) \end{array}\right.$$ (25)

Subsequently, we use the gradient descent algorithm to minimize the above estimation error, thereby obtaining the estimated direction vector ${\hat{r}}_{\mathrm{ori}}$ and giving its update rule as follows:

$${\hat{\dot{r}}}_{\mathrm{ori}}=\rho R_{o}^{\text{T}}e_{i}-\eta r_{\mathrm{ori}},$$ (26)

where ρ and η are both positive constants, and we obtain in Equation 27

$$\left\{\begin{array}{cc} & {\hat{x}}_{o1}=x_{c1}-R_{o}{\hat{r}}_{\mathrm{or1}}\\ & {\hat{x}}_{o2}=x_{c2}-R_{o}{\hat{r}}_{\mathrm{or2}} \end{array}\right.$$ (27)

According to the above formula, the estimated value of the COM position of the target object is obtained as Equation 28 ${\hat{x}}_{o}\left(k\right)=\frac{1}{2}\sum_{i=1}^{2}{\hat{x}}_{oi}\left(k\right)$ . Through continuous iteration, the estimated value of the target tool’s COM position can ultimately be approximated to a small neighborhood of its true value. The following is a convergence analysis of the object COM position estimator based on the gradient model designed in this section:

First, define a cost function $E_{i}=\frac{1}{2}e_{i}^{\text{T}}e_{i}$ . According to the Newton–Raphson algorithm, when the update rule for the estimated direction vector ${\hat{r}}_{\mathrm{ori}}$ is a partial derivative of the cost function to ${\hat{r}}_{\mathrm{ori}}$ , $E_{i}$ , and $e_{i}$ can be minimized, thereby obtaining as Equation 28

$$\begin{array}{cc} & {\hat{\dot{r}}}_{\mathrm{ori}}=-\rho \frac{\partial E_{i}}{\partial {\hat{r}}_{\mathrm{ori}}}=-\rho \frac{\partial e_{i}^{\text{T}}e_{i}}{2\partial {\hat{r}}_{\mathrm{ori}}}\\ & \;=-\rho \frac{\partial e_{i}^{\text{T}}}{\partial {\hat{r}}_{\mathrm{ori}}}e_{i}=-\rho \frac{\partial \left(x_{ci}-{\hat{x}}_{oi}-R_{o}{\hat{r}}_{\mathrm{ori}}\right)}{\partial {\hat{r}}_{\mathrm{ori}}}e_{i}\\ & \;=\rho R_{o}^{\text{T}}e_{i}. \end{array}$$ (28)

The term $\eta r_{\mathrm{ori}}$ is added to Equation 26 to increase the convergence velocity of the estimation error, and the analysis is completed.

### 2.5 Dynamic analysis based on the object’s center of mass estimation

Combining the estimated grasping matrix ${\hat{J}}_{oi}^{\text{T}}$ , the dynamic equation of a single manipulator combined with an object can be expressed as in Equations 29, 30:

$$\begin{array}{cc} u_{i} & =\left[I_{mi}{\gamma_{i}}^{2}+\hat{\Gamma_{i}}M_{D}\left(x_{o}\right){\hat{J}}_{\phi i}\right]{\ddot{q}}_{i}+\left[\hat{\Gamma_{i}}M_{D}\left(x_{o}\right){\dot{\hat{J}}}_{\phi i}+\hat{\Gamma_{i}}C_{D}\left(x_{o},{\dot{x}}_{o}\right){\hat{J}}_{\phi i}\right]{\dot{q}}_{i}\\ & \;+F_{i}+\tau_{Si}+\tau_{i}+\hat{\Gamma_{i}}g_{D}\left(x_{o}\right)+\hat{\Gamma_{i}}{\hat{e}}_{Mi}+e_{Ji}, \end{array}$$ (29)

$$e_{Ji}=\frac{1}{2}J_{si}^{\text{T}}{\left({\tilde{J}}_{oi}^{\text{T}}\right)}^{+}\left[M_{D}{\hat{J}}_{\phi i}{\ddot{q}}_{i}+\left(M_{D}{\dot{\hat{J}}}_{\phi i}+C_{D}{\hat{J}}_{\phi i}\right){\dot{q}}_{i}+g_{D}+{\hat{e}}_{Mi}\right],$$ (30)

where $\hat{\Gamma }=\frac{1}{2}J_{si}^{\text{T}}{\left({\hat{J}}_{oi}^{\text{T}}\right)}^{+}$ , ${\hat{J}}_{\phi i}={\hat{J}}_{oi}^{+}J_{si}$ . $e_{Ji}$ is the error compensation term estimated by the grasping matrix.

Based on Equation 29, the state space equation of the dynamic of the DRM system can be described as follows:

$$\left\{\begin{array}{cc} & {\dot{x}}_{1i}\left({\hat{J}}_{\phi i}\right)=x_{2i}\left({\hat{J}}_{\phi i}\right)\\ & {\dot{x}}_{2i}\left({\hat{J}}_{\phi i}\right)={\hat{▵}}_{Di}+{\hat{N}}_{Di}f_{ai}+{\hat{N}}_{Di}\left(u_{i}-\tau_{Si}-\tau_{i}\right), \end{array}\right.$$ (31)

where $x_{2i}\left({\hat{J}}_{\phi i}\right)$ denotes the system state of the grasping matrix following substitution estimation.

${\hat{▵}}_{Di}=-{\left[I_{mi}{\gamma_{i}}^{2}+\hat{\Gamma_{i}}M_{D}\left(x_{o}\right){\hat{J}}_{\phi i}\right]}^{-1}\left[\left(\hat{\Gamma_{i}}M_{D}\left(x_{o}\right)\right.\right.{\dot{\hat{J}}}_{\phi i}+\hat{\Gamma_{i}}C_{D}\left(x_{o},{\dot{x}}_{o}\right)$ $\left.{\hat{J}}_{\phi i}\right){\dot{q}}_{i}+\hat{\Gamma_{i}}g_{D}\left.\left(x_{o}\right)\right]$ ${\hat{N}}_{Di}={\left[I_{mi}{\gamma_{i}}^{2}+\hat{\Gamma_{i}}M_{D}\left(x_{o}\right){\hat{J}}_{\phi i}\right]}^{-1}$ , $f_{ai}=-F_{i}-\hat{\Gamma_{i}}{\hat{e}}_{Mi}-e_{Ji}$ is an unknown term.

## 3 Design of the ADP-based coordinated control scheme

### 3.1 Derivation of optimal coordinated control

Define a reference desired velocity ${\dot{q}}_{\mathrm{ijd}}$ in Equation 32:

$${\dot{q}}_{\mathrm{ijd}}={{\hat{J}}^{+}}_{\phi i}\left[{\dot{x}}_{od}-\beta \left(x_{o}-x_{od}\right)\right],$$ (32)

where $x_{od}$ is the desired position of the object; ${\dot{x}}_{od}$ is the desired velocity; and β is a positive constant.

Define the joint racking error in Equation 33:

$$e_{qi}={{\hat{J}}^{+}}_{\phi i}\left(x_{o}-x_{od}\right)=q_{i}-q_{\mathrm{ijd}}.$$ (33)

$F_{Ii}$ can be parameterized by the vector of the Lagrange multiplier as in Equation 34:

$$F_{Ii}=\xi^{\text{T}}\lambda_{Ii},$$ (34)

where $\xi^{\text{T}}$ is the direction vector of the internal force and $\lambda_{Ii}$ represents the magnitude of the internal force.

To ensure that the joint tracking error $e_{qi}$ and the internal force error $e_{\lambda_{Ii}}$ remain sufficiently small to obtain optimal control $u_{i}^{*}$ , the deviation of the fusion performance index function is in Equation 36

$$\underset{u_{i}\in \Psi \left(\Omega \right)}{J_{i}\left(s_{i}\right)}=\int_{t}^{\infty }\left(N_{i}\left(s_{i},u_{i}\right)\right)d\tau ,$$ (35)

$$s_{i}\left(\tau_{i}\right)=K_{dj}{\dot{e}}_{qi}+K_{qj}e_{qi}+K_{cj}\int_{0}^{t_{k}}e_{\lambda_{Ii}}d\tau ,$$ (36)

where $N_{i}\left(s_{i},u_{i}\right)=s_{i}^{\text{T}}Q_{i}s_{i}+u_{i}^{\text{T}}R_{i}u_{i}$ denotes the utility function. $s_{i}\left(\tau_{i}\right)$ is the tracking error fusion function with ${\dot{e}}_{qi}={\dot{q}}_{i}-{\dot{q}}_{\mathrm{ijd}}$ , $K_{dj},K_{qj},K_{cj}$ are control parameters, and the initial state of this function is $s_{0}\left(\tau_{i}\right)=s_{i}\left(0\right)$ . $Q_{i}$ , $R_{i}$ are determined positive definite matrices. $\Psi \left(\Omega \right)$ are the admissible control policy set.

Definition 1: The control strategy $u_{i}$ is admissible via the performance index function (Equation 35), if $u_{i}$ stabilizes the DRM (Equation 11) on the compact set C and $J_{i}$ is finite for ∀x∈C .

Based on Equation 35, the Hamilton function is

$$\begin{array}{cc} H_{i}\left(s_{i},u_{i},\nabla J_{i}\right) & =N_{i}\left(s_{i},u_{i}\right)+{\left(\nabla J_{i}\right)}^{\text{T}}\cdot {\dot{s}}_{i}\\ & =s_{i}^{\text{T}}Q_{i}s_{i}+u_{i}^{\text{T}}R_{i}u_{i}+{\left(\nabla J_{i}\right)}^{\text{T}}\\ & \;\cdot \left(K_{dj}\left({\hat{▵}}_{Di}+{\hat{N}}_{Di}f_{ai}+{\hat{N}}_{Di}\left(u_{i}-\tau_{Si}-\tau_{i}\right)\right)+v_{ji}\right), \end{array}$$ (37)

where $\nabla J_{i}\left(s_{i}\right)=\partial J_{i}\left(s_{i}\right)/\partial s_{i},v_{ji}=-K_{dj}{\ddot{q}}_{\mathrm{ijd}}+K_{qj}{\dot{e}}_{qi}+K_{cj}e_{\lambda_{Ii}}$ . Define the optimal performance index function:

$$J_{i}^{*}\left(s_{i}\right)=\underset{u_{i}}{\min}\;\int_{t}^{\infty }N_{i}\left(s_{i},u_{i}\right)d\tau .$$ (38)

Combining the above Hamilton function (Equation 37) and the optimal performance index function (Equation 38) and applying optimization principles, the optimal performance index function satisfies the HJB equation as Equation 39:

$$0=\underset{u_{i}}{\min}\;H_{i}\left(s_{i},u_{i},\nabla J_{i}^{*}.\right)$$ (39)

If $J_{i}^{*}$ exists and is continuously differentiable, we can obtain the optimal control law as follows:

$$u_{i}^{*}=-\frac{1}{2}K_{dj}R_{i}^{-1}{\hat{N}}_{Di}^{\text{T}}\nabla J_{i}^{*}\left(s_{i}\right).$$ (40)

Next, employ the policy iteration (PI) algorithm to identify the optimal control strategy. The specific steps are as follows:

Input: k=0 , initial feasible control $u_{i}^{\left(0\right)}$ , and a small positive constant $\varsigma_{i}$ ;

1) Solve $J_{i}^{\left(k\right)}$ from $0=N_{i}\left(s_{i},u_{i}^{\left(k\right)}\right)+{\left(\nabla J_{i}^{\left(k+1\right)}\right)}^{\text{T}}\cdot \left(K_{dj}\left({\hat{▵}}_{Di}+{\hat{N}}_{Di}f_{ai}\right.\right.$ $+{\hat{N}}_{Di}\left.\left.\left(u_{i}^{\left(k\right)}-\tau_{Si}-\tau_{i}\right)\right)+v_{ji}\right)$ , with $J_{i}^{\left(k+1\right)}\left(0\right)=0$ .

2) Update $u_{i}^{\left(k\right)}$ via $u_{i}^{\left(k+1\right)}=-\frac{1}{2}K_{dj}R_{i}^{-1}{\hat{N}}_{Di}^{\text{T}}\nabla J_{i}^{\left(k+1\right)}$ .

If $\left\|J_{i}^{\left(k+1\right)}-J_{i}^{\left(k\right)}\right\|\leq \varsigma_{i}$ then stop and obtain the approximate optimal control.

Else let k=k+1 and return to procedure 1.

Output: The optimal solution $u_{i}^{*}$ .

Through iterative computation of this algorithm, as the iteration index k→∞ , the optimal performance index function and optimal control strategy can be approximated.

### 3.2 Adaptive observer design

Next, we approximate the unknown model term in real time by designing an adaptive observer:

$$\left\{\begin{array}{cc} & {\dot{\hat{x}}}_{1i}={\hat{x}}_{2i}+k_{1i}\left(x_{1i}-{\hat{x}}_{1i}\right)\\ & {\dot{\hat{x}}}_{2i}={\hat{▵}}_{Di}+{\hat{N}}_{Di}{\hat{f}}_{ai}+{\hat{N}}_{Di}\left(u_{i}-\tau_{Si}-\tau_{i}\right)+k_{2i}\left(x_{2i}-{\hat{x}}_{2i}\right) \end{array}\right.$$ (41)

where ${\hat{x}}_{i}={\left[{\hat{x}}_{1},{\hat{x}}_{2}\right]}^{\text{T}}$ is the observed value of the state of the reconfigurable manipulator system; $k_{1i}$ and $k_{2i}$ are positive gains given by the observer; and $e_{Fi}={\left[E_{1i},E_{2i}\right]}^{\text{T}}={\left[x_{1i}-{\hat{x}}_{1i},x_{2i}-{\hat{x}}_{2i}\right]}^{\text{T}}$ represents the system state observation error. Combining Equation 31 and Equation 41, we obtain as Equation 42

$${\dot{e}}_{Fi}=\left[\begin{array}{c} {\dot{E}}_{1i}\\ {\dot{E}}_{2i} \end{array}\right]\begin{array}{c} =\\ = \end{array}\left[\begin{array}{c} {\dot{x}}_{1i}-{\dot{\hat{x}}}_{1i}\\ {\dot{x}}_{2i}-{\dot{\hat{x}}}_{2i} \end{array}\right]\begin{array}{c} =\\ = \end{array}\left[\begin{array}{c} E_{2i}-k_{1i}E_{1i}\\ {\hat{N}}_{Di}e_{fi}-k_{2i}E_{2i} \end{array}\right],$$ (42)

where $e_{fi}=f_{ai}-{\hat{f}}_{ai}$ denotes the observational error of the model uncertainty term.

Estimation of model uncertainty can be updated in Equation 43:

$${\dot{\hat{f}}}_{ai}=\alpha_{Fi}{\hat{N}}_{Di}E_{2i},$$ (43)

where $\alpha_{Fi}$ is a positive constant.

Theorem 1. Consider the adaptive observer (Equation 41) approximating the unknown model terms in Equation 31. The observation error is uniformly ultimately bounded (UUB) based on the adaptive update strategy (Equation 43).

Proof. As follows, the candidate Lyapunov function is defined:

$$V_{1i}\left(t\right)=\frac{1}{2}e_{Fi}^{\text{T}}e_{Fi}+\frac{1}{2}e_{fi}^{\text{T}}\alpha_{Fi}^{-1}e_{fi}.$$ (44)

The derivative of Equation 44 with respect to time is

$$\begin{array}{cc} {\dot{V}}_{1i}\left(t\right) & =e_{Fi}^{\text{T}}{\dot{e}}_{Fi}+e_{fi}^{\text{T}}\alpha_{Fi}^{-1}{\dot{e}}_{fi}\\ & =E_{1i}{\dot{E}}_{1i}+E_{2i}{\dot{E}}_{2i}-{\dot{\hat{f}}}_{i}^{\text{T}}\alpha_{Fi}^{-1}e_{fi}\\ & =E_{1i}\left(E_{2i}-k_{1i}E_{1i}\right)+E_{2i}\left({\hat{N}}_{Di}e_{fi}-k_{2i}E_{2i}\right)-{\dot{\hat{f}}}_{i}^{\text{T}}\alpha_{Fi}^{-1}e_{fi}\\ & =E_{1i}E_{2i}-k_{1i}E_{1i}^{2}-k_{2i}E_{2i}^{2}+\left({\hat{N}}_{Di}E_{2i}-{\dot{\hat{f}}}_{i}^{\text{T}}\alpha_{Fi}^{-1}\right)e_{fi}\\ & \leq -\left(k_{1i}-\frac{1}{2}\right)E_{1i}^{2}-\left(k_{2i}-\frac{1}{2}\right)E_{2i}^{2}-\left({\dot{\hat{f}}}_{i}^{\text{T}}\alpha_{Fi}^{-1}-{\hat{N}}_{Di}E_{2i}\right)e_{fi}. \end{array}$$ (45)

Substitute the updated law Equation 40 into Equation 45. When $k_{1i}\geq \frac{1}{2}$ and $k_{2i}\geq \frac{1}{2}$ are satisfied, ${\dot{V}}_{1i}\left(t\right)\leq 0$ . Therefore, the observational error of the above model is UUB, which completes the proof.

### 3.3 Critic neural network implementation

Combining Equations 35, 41, we obtain the Hamiltonian function as Equation 46:

$$\begin{array}{cc} H_{i}\left({\hat{s}}_{i},u_{i},\nabla J_{i}\right) & =N_{i}\left({\hat{s}}_{i},u_{i}\right)+{\left(\nabla J_{i}\right)}^{\text{T}}\cdot {\dot{\hat{s}}}_{i}\\ & =N_{i}\left({\hat{s}}_{i},u_{i}\right)+{\left(\nabla J_{i}\right)}^{\text{T}}\\ & \;\cdot \left(K_{dj}\left({\hat{▵}}_{Di}+{\hat{N}}_{Di}f_{ai}+{\hat{N}}_{Di}\left(u_{i}-\tau_{Si}-\tau_{i}\right)\right)+v_{ji}\right). \end{array}$$ (46)

To obtain the optimal control strategy, the Hamiltonian function (Equation 46) should be solved to obtain the optimal performance index function $J_{i}^{*}$ . The optimal control strategy is then obtained through the PI algorithm and the approximation ability of the neural network. Therefore, a critic neural network is constructed in Equation 47:

$$J_{i}\left({\hat{s}}_{i}\right)=W_{ij}^{\text{T}}\delta_{ij}\left({\hat{s}}_{i}\right)+\varepsilon_{ij},$$ (47)

where $W_{ij}\in R^{N_{i}}$ denotes the ideal weight of neural network, $N_{i}$ is the number of hidden neurons, $\delta_{ij}\left({\hat{s}}_{i}\right)$ is the active function, and $\varepsilon_{ij}$ is the critic NN approximation error.

Therefore, the partial derivative of $J_{i}\left({\hat{s}}_{i}\right)$ is expressed as in Equation 48:

$$\nabla J_{i}\left({\hat{s}}_{i}\right)={\left(\nabla \delta_{ij}\left({\hat{s}}_{i}\right)\right)}^{\text{T}}W_{ij}+\nabla \varepsilon_{ij},$$ (48)

where $\nabla \delta_{ij}\left({\hat{s}}_{i}\right)=\partial \delta_{ij}\left({\hat{s}}_{i}\right)/\partial {\hat{s}}_{i}$ and $\nabla \varepsilon_{ij}$ are the partial derivatives of the active function and the critic NN approximation error. Combining Equation 46 and Equation 48 yields

$$\begin{array}{cc} H_{i}\left({\hat{s}}_{i},u_{i},W_{ij}\right) & =N_{i}\left({\hat{s}}_{i},u_{i}\right)+{\left({\left(\nabla \delta_{ij}\left({\hat{s}}_{i}\right)\right)}^{\text{T}}W_{ij}+\nabla \varepsilon_{ij}\right)}^{\text{T}}\cdot {\dot{\hat{s}}}_{i}\\ & =N_{i}\left({\hat{s}}_{i},u_{i}\right)+\left(W_{ij}^{\text{T}}\nabla \delta_{ij}\left({\hat{s}}_{i}\right)+\nabla \varepsilon_{ij}^{\text{T}}\right)\\ & \;\cdot \left(K_{dj}\left({\hat{▵}}_{Di}+{\hat{N}}_{Di}{\hat{f}}_{ai}+{\hat{N}}_{Di}\left(u_{i}-\tau_{Si}-\tau_{i}\right)\right)+v_{ji}\right)\\ & =-\nabla \varepsilon_{ij}^{\text{T}}{\dot{\hat{s}}}_{i}=e_{\mathrm{ijh}}, \end{array}$$ (49)

where $e_{\mathrm{ijh}}$ is the residual obtained by approximating the HJB function using an ideal critic neural network. Because we cannot directly obtain the ideal critic neural network weight $W_{ij}$ , we approximate the critic neural network as in Equation 50:

$${\hat{J}}_{i}\left({\hat{s}}_{i}\right)={\hat{W}}_{ij}^{\text{T}}\delta_{ij}\left({\hat{s}}_{i}\right),$$ (50)

where ${\hat{W}}_{ij}$ is the approximate value of the neural network weight. Combining Equation 49, $\nabla {\hat{J}}_{i}\left(s_{i}\right)$ is expressed as follows:

$$\nabla {\hat{J}}_{i}\left({\hat{s}}_{i}\right)={\left(\nabla \delta_{ij}\left({\hat{s}}_{i}\right)\right)}^{\text{T}}{\hat{W}}_{ij}.$$ (51)

Therefore, by utilizing Equations 49, 51, we can derive the approximate Hamiltonian function:

$$\begin{array}{cc} & {\hat{H}}_{i}\left({\hat{s}}_{i},u_{i},{\hat{W}}_{ij}\right)=N_{i}\left({\hat{s}}_{i},u_{i}\right)+{\left({\left(\nabla \delta_{ij}\left({\hat{s}}_{i}\right)\right)}^{\text{T}}{\hat{W}}_{ij}\right)}^{\text{T}}\cdot {\dot{\hat{s}}}_{i}\\ & \;=N_{i}\left({\hat{s}}_{i},u_{i}\right)+\left({\hat{W}}_{ij}^{\text{T}}\nabla \delta_{ij}\left({\hat{s}}_{i}\right)\right)\\ & \;\cdot \left(K_{dj}\left({\hat{▵}}_{Di}+{\hat{N}}_{Di}{\hat{f}}_{ai}+{\hat{N}}_{Di}\left(u_{i}-\tau_{Si}-\tau_{i}\right)\right)+v_{ji}\right)\\ & \;=e_{ij}, \end{array}$$ (52)

where $e_{ij}$ is the estimated approximate error of the Hamiltonian function. Next, we minimize objective function to adjust the neural network weight vector $E_{ij}=\frac{1}{2}e_{ij}^{\text{T}}e_{ij}$ using the gradient descent algorithm and design its update strategy as follows:

$${\dot{\hat{W}}}_{ij}=-\alpha_{ij}e_{ij}\nabla \delta_{ij}\left({\hat{s}}_{i}\right){\dot{\hat{s}}}_{i},$$ (53)

where $\alpha_{ij}$ is learning rate of critic NN. Define $v_{i}=\nabla \delta_{ij}\left({\hat{s}}_{i}\right){\dot{\hat{s}}}_{i}$ . Suppose that $v_{i}$ is bounded and there exists a positive constant $v_{ai}$ such that $\left\|v_{i}\right\|\leq v_{ai}$ holds.

The estimation error associated with the weights of the critic neural network is formulated as follows:

$${\tilde{W}}_{ij}=W_{ij}-{\hat{W}}_{ij}.$$ (54)

Combining Equations 52–54, we obtain

$$e_{ij}=e_{\mathrm{ijh}}-{\tilde{W}}_{ij}^{\text{T}}v_{i}.$$ (55)

Combining the critic neural network update rule (Equation 53) with Equations 54, 55, we obtain the update rule for the weight estimation error as in Equation 56:

$${\dot{\tilde{W}}}_{ij}=-{\dot{\hat{W}}}_{ij}=\alpha_{ij}e_{ij}v_{i}=\alpha_{ij}\left(e_{\mathrm{ijh}}-{\tilde{W}}_{ij}^{\text{T}}v_{i}\right)v_{i}.$$ (56)

The final approximate optimal control law is in Equation 57

$${\hat{u}}_{i}^{*}=-\frac{1}{2}K_{dj}{R_{i}}^{-1}{\hat{N}}_{Di}^{\text{T}}{\left(\nabla \delta_{ij}\left({\hat{s}}_{i}\right)\right)}^{\text{T}}{\hat{W}}_{ij}.$$ (57)

### 3.4 Proof of stability

The stability of the developed optimal control for reconfigurable manipulators is analyzed using Lyapunov functions.

Provided that the persistent excitation condition holds, the weight vector can be iteratively refined until it converges toward its optimal value.

Theorem 2. The dynamic model of the DRM system and the object are illustrated in Equation 11 and Equation 12, and the state space equation is formulated in Equation 31. The critic NN weight approximation error, the internal force error, and the position tracking error are UUB via the presented distributed coordinated optimal control derived in Equation 57 via the weight update strategy (Equation 53).

Proof. Select the Lyapunov candidate function as in Equation 58

$$V\left(t\right)=\overset{2}{\underset{i=1}{\sum }}V_{i}\left(t\right)=\overset{2}{\underset{i=1}{\sum }}\left(\frac{1}{2}{s_{i}}^{\text{T}}s_{i}+J_{i}^{*}\left(s_{i}\right)+\frac{1}{2\alpha_{ij}}{\tilde{W}}_{ij}^{\text{T}}{\tilde{W}}_{ij}\right).$$ (58)

The time derivative of $V\left(t\right)$ as Equation 59

$$\begin{array}{cc} & \dot{V}\left(t\right)=\overset{2}{\underset{i=1}{\sum }}{\dot{V}}_{i}\left(t\right)\\ & \;=\overset{2}{\underset{i=1}{\sum }}\left(s_{i}^{\text{T}}{\dot{s}}_{i}+{\left(\nabla J_{i}^{*}\left(s_{i}\right)\right)}^{\text{T}}{\dot{s}}_{i}+\frac{1}{\alpha_{ij}}{\tilde{W}}_{ij}^{\text{T}}{\dot{\tilde{W}}}_{ij}\right)\\ & \;=\overset{2}{\underset{i=1}{\sum }}\left(\begin{array}{cc} & s_{i}^{\text{T}}\left(K_{dj}\left({\hat{▵}}_{Di}+{\hat{N}}_{Di}{\hat{f}}_{ai}+{\hat{N}}_{Di}\left(u_{i}-\tau_{Si}-\tau_{i}\right)\right)+v_{ji}\right)\\ & \;+{\left(\nabla J_{i}^{*}\left(s_{i}\right)\right)}^{\text{T}}{\dot{s}}_{i}+\frac{1}{\alpha_{ij}}{\tilde{W}}_{ij}^{\text{T}}{\dot{\tilde{W}}}_{ij} \end{array}\right)\\ & \;=\overset{2}{\underset{i=1}{\sum }}\left(\begin{array}{cc} & s_{i}^{\text{T}}\left(K_{dj}\left({\hat{▵}}_{Di}+{\hat{N}}_{Di}{\hat{f}}_{ai}+{\hat{N}}_{Di}\left(u_{i}-\tau_{Si}-\tau_{i}\right)\right)+v_{ji}\right)\\ & \;-s_{i}^{\text{T}}Q_{i}s_{i}-u_{i}^{\text{T}}R_{i}u_{i}+{\tilde{W}}_{ij}^{\text{T}}\left(e_{\mathrm{ijh}}-{\tilde{W}}_{ij}^{\text{T}}v_{i}\right)v_{i} \end{array}\right)\\ & \;=\overset{2}{\underset{i=1}{\sum }}\left(\begin{array}{cc} & s_{i}^{\text{T}}\left(K_{dj}\left({\hat{▵}}_{Di}+{\hat{N}}_{Di}{\hat{f}}_{ai}+{\hat{N}}_{Di}\left(u_{i}-\tau_{Si}-\tau_{i}\right)\right)+v_{ji}\right)\\ & \;-s_{i}^{\text{T}}Q_{i}s_{i}-u_{i}^{\text{T}}R_{i}u_{i}+{\tilde{W}}_{ij}^{\text{T}}e_{\mathrm{ijh}}v_{i}-{\left\|{\tilde{W}}_{ij}^{\text{T}}v_{i}\right\|}^{2} \end{array}\right). \end{array}$$ (59)

According to the properties of reconfigurable manipulator, there exist positive parameters $L_{if}$ , $w_{ig}$ , and $L_{il}$ such that inequalities $\left\|{\hat{▵}}_{Di}\right\|\leq L_{if}\left\|s_{i}\right\|$ , $\left\|{\hat{N}}_{Di}\right\|\leq w_{ig}$ , and $\left\|{\hat{f}}_{ai}\right\|\leq L_{il}$ hold. Combining these with Young’s inequality, the above equation can be derived as Equation 60:

$$\begin{array}{cc} & \dot{V}\left(t\right)\leq \overset{2}{\underset{i=1}{\sum }}\left(\begin{array}{cc} & K_{dj}L_{if}{\left\|s_{i}\right\|}^{2}+K_{dj}w_{ig}L_{il}\left\|s_{i}\right\|+K_{dj}w_{ig}\left\|s_{i}\right\|\left\|u_{i}\right\|\\ & -K_{dj}w_{ig}\tau_{Si}\left\|s_{i}\right\|-K_{dj}w_{ig}\tau_{i}\left\|s_{i}\right\|+v_{ij}\left\|s_{i}\right\|\\ & -s_{i}^{\text{T}}Q_{i}s_{i}-u_{i}^{\text{T}}R_{i}u_{i}+{\tilde{W}}_{ij}^{\text{T}}e_{\mathrm{ijh}}v_{i}-{\left\|{\tilde{W}}_{ij}^{\text{T}}v_{i}\right\|}^{2} \end{array}\right)\\ & \;\leq \overset{2}{\underset{i=1}{\sum }}\left(\begin{array}{cc} & K_{dj}L_{if}{\left\|s_{i}\right\|}^{2}+K_{dj}w_{ig}L_{il}\left\|s_{i}\right\|+\frac{1}{2}{K_{dj}}^{2}{\left\|s_{i}\right\|}^{2}\\ & +\frac{1}{2}{w_{ig}}^{2}{\left\|u_{i}\right\|}^{2}-K_{dj}w_{ig}\tau_{Si}\left\|s_{i}\right\|-K_{dj}w_{ig}\tau_{i}\left\|s_{i}\right\|+v_{ij}\left\|s_{i}\right\|\\ & -\lambda_{\min}\left(Q_{i}\right){\left\|s_{i}\right\|}^{2}-\lambda_{\min}\left(R_{i}\right){\left\|u_{i}\right\|}^{2}+\frac{1}{2}{\left\|e_{\mathrm{ijh}}\right\|}^{2}-\frac{1}{2}{\left\|{\tilde{W}}_{ij}^{\text{T}}v_{i}\right\|}^{2} \end{array}\right)\\ & \;\leq \overset{2}{\underset{i=1}{\sum }}\left(\begin{array}{cc} & -\left(\lambda_{\min}\left(Q_{i}\right)-K_{dj}L_{if}-\frac{1}{2}{K_{dj}}^{2}\right){\left\|s_{i}\right\|}^{2}\\ & -\left(-K_{dj}w_{ig}L_{il}+K_{dj}w_{ig}\tau_{Si}+K_{dj}w_{ig}\tau_{i}-v_{ij}\right)\left\|s_{i}\right\|\\ & -\left(\lambda_{\min}\left(R_{i}\right)-\frac{1}{2}{w_{ig}}^{2}\right){\left\|u_{i}\right\|}^{2}-\frac{1}{2}\left({\left\|{\tilde{W}}_{ij}^{\text{T}}v_{i}\right\|}^{2}-{\left\|e_{\mathrm{ijh}}\right\|}^{2}\right) \end{array}\right). \end{array}$$ (60)

The angular acceleration ${\ddot{q}}_{\mathrm{ijd}}$ , position tracking error $e_{qi}$ of the known desired trajectory, and internal force error $e_{\lambda_{Ii}}$ are bounded, and $\left\|{\ddot{q}}_{\mathrm{ijd}}\right\|\leq \sigma_{i}$ , $\left\|e_{qi}\right\|\leq \xi_{i}$ , and $\left\|e_{\lambda_{Ii}}\right\|\leq \eta_{i}$ exist, where $\sigma_{i}$ , $\xi_{i}$ , and $\eta_{i}$ are upper bound normal numbers. The above equation can be summarized as in Equation 61:

$$\dot{V}\left(t\right)\leq \overset{2}{\underset{i=1}{\sum }}\left(\begin{array}{cc} & -\left(\Phi_{i1}\left\|s_{i}\right\|+\left(-K_{dj}w_{ig}L_{il}+K_{dj}w_{ig}\tau_{Si}+K_{dj}w_{ig}\tau_{i}-v_{ij}\right)\right)\left\|s_{i}\right\|\\ & -\Phi_{i2}{\left\|u_{i}\right\|}^{2}-\frac{1}{2}\left({\left\|{\tilde{W}}_{ij}^{\text{T}}v_{i}\right\|}^{2}-{\left\|e_{\mathrm{ijh}}\right\|}^{2}\right), \end{array}\right)$$ (61)

where $\Phi_{i1}=\lambda_{\min}\left(Q_{i}\right)-K_{dj}L_{if}-\frac{1}{2}{K_{dj}}^{2}$ and $\Phi_{i2}=\lambda_{\min}\left(R_{i}\right)-\frac{1}{2}{w_{ig}}^{2}$ .

Therefore, we can conclude that when $s_{i}$ satisfies outside $\Omega_{i1}=\left\{s_{i}:\left\|s_{i}\right\|\leq \frac{K_{dj}w_{ig}L_{il}-K_{dj}w_{ig}\tau_{Si}-K_{dj}w_{ig}\tau_{i}+K_{dj}\sigma_{i}+K_{qj}\xi_{i}+K_{cj}\eta_{i}}{\Phi_{i1}}\right\}$ and $W_{ij}$ satisfies outside $\Omega_{i2}=\left\{{\tilde{W}}_{ij}:\left\|{\tilde{W}}_{ij}\right\|\leq \left\|\frac{e_{\mathrm{ijh}}}{v_{i}}\right\|\right\}$ , then $\dot{V}\left(t\right)\leq 0$ can be guaranteed. The parameters must satisfy the following conditions as in Equation 62:

$$\left\{\begin{array}{cc} & \lambda_{\min}\left(Q_{i}\right)\geq K_{dj}L_{if}+\frac{1}{2}{K_{dj}}^{2}\\ & \lambda_{\min}\left(R_{i}\right)\geq \frac{1}{2}{w_{ig}}^{2} \end{array}\right.$$ (62)

Through the above stability proof and analysis, under the designed control strategy, the tracking error and the internal force tracking error of the dual-arm reconfigurable manipulator can be UUB.

## 4 Experimental study

### 4.1 Experiment settings

**FIGURE 1:**
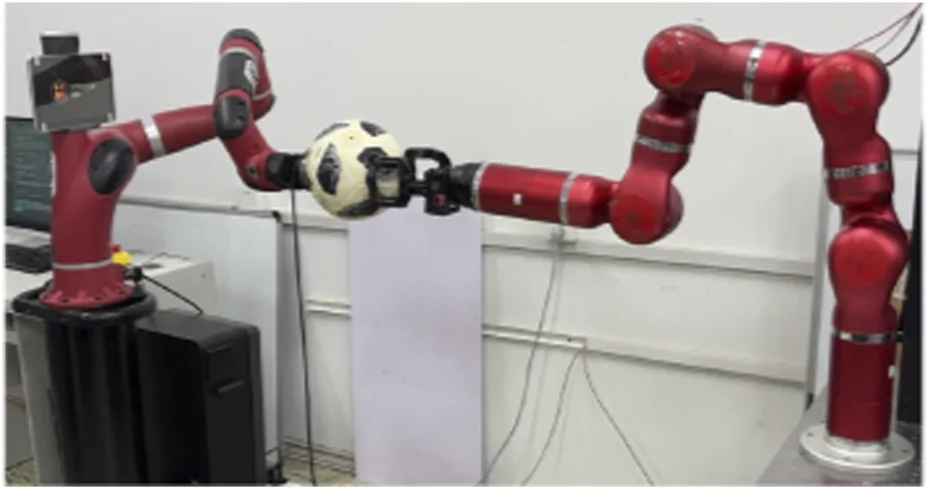
Dual-arm reconfigurable manipulator platform.

Distributed coordinated optimal control is experimentally verified using a DRM system that consists of a five-degree-of-freedom (5-DoF) reconfigurable manipulator and a 7-DoF manipulator (cf Figure 1). Two reconfigurable manipulators jointly perform the experiment of object grasping, and the trajectory of the target tool is a reciprocating linear motion along the Z-axis. The critic NN is chosen as the radial basis function neural network (RBFNN), and the activation function of Equation 47 is $\delta_{ij}=e^{\frac{-{\left({\dot{\hat{s}}}_{i}-\zeta_{i}\right)}^{T}\left({\dot{\hat{s}}}_{i}-\zeta_{i}\right)}{\ell_{i}}}$ with certain parameters $\zeta_{i}$ as well as $\ell_{i}$ . The learning rate of the critic neural network can yield solutions that diverge from the optimal control result. When parameter $\alpha_{ij}$ is set too small, overtraining occurs. The iterative process becomes excessively time-consuming, while the performance index improves only marginally. In robotic systems, fast response is as critical a performance index as accuracy. Conversely, selecting an excessively large learning rate leads to undertraining, which compromises tracking accuracy. Based on various experimental investigations, an appropriate learning rate is chosen as $\alpha_{ij}=0.85$ . The effectiveness of the proposed method is verified through the position tracking in Cartesian space and joint space error, as well as the control torque and NN weight compared with the existing learning-based optimal method (Qin et al., 2023).

### 4.2 Experiment analysis

**FIGURE 2:**
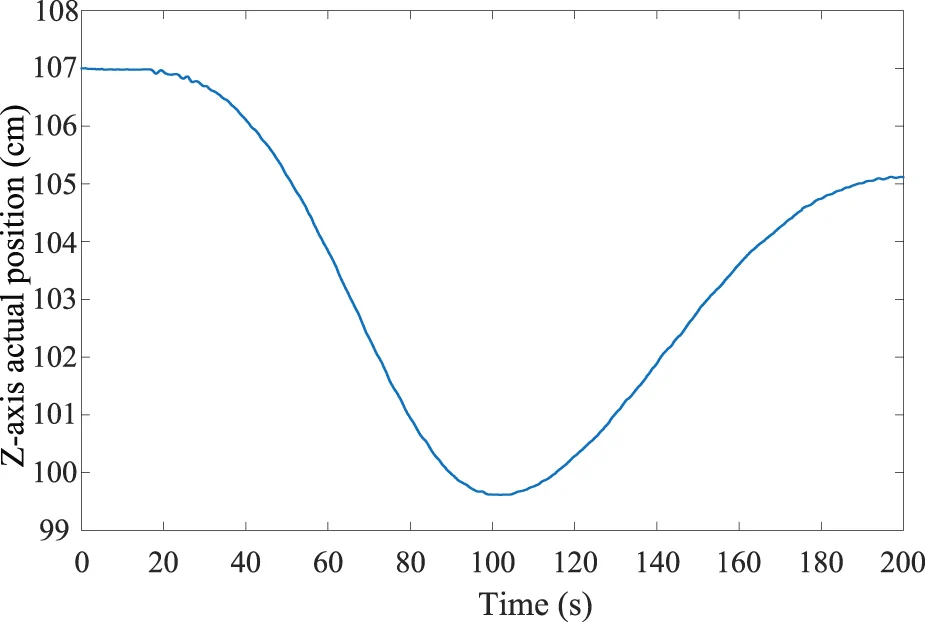
Proposed method Z-axis actual position.

**FIGURE 3:**
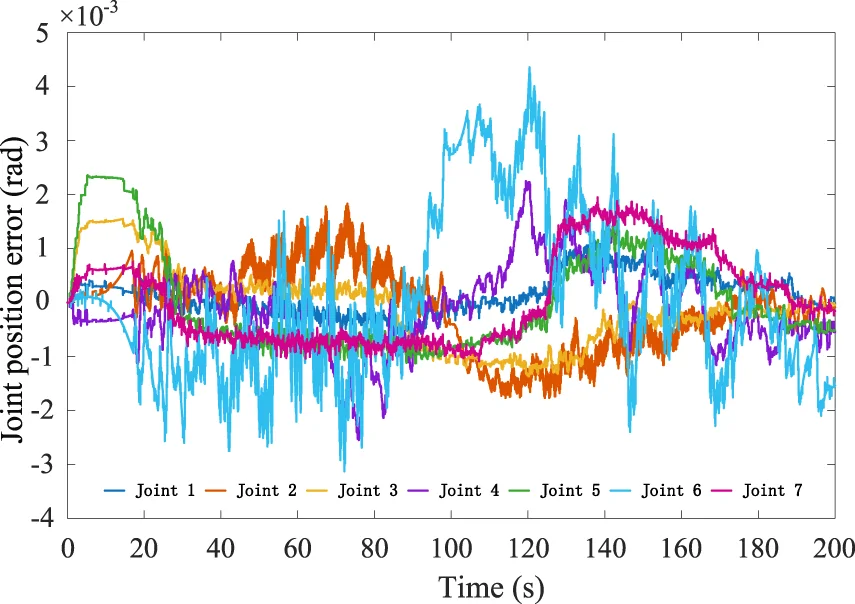
Manipulator 1 existing method joint position error.

**FIGURE 4:**
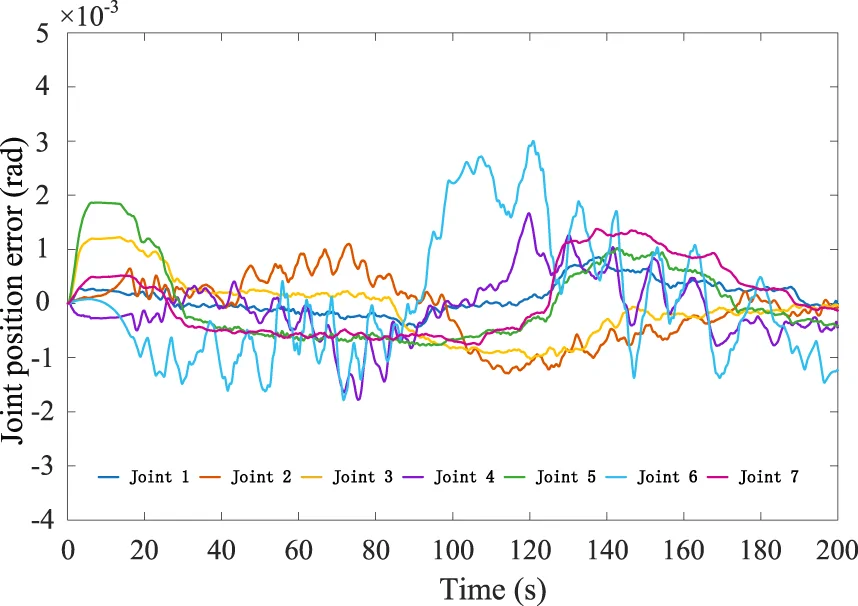
Manipulator 1 proposed method joint position error.

**FIGURE 5:**
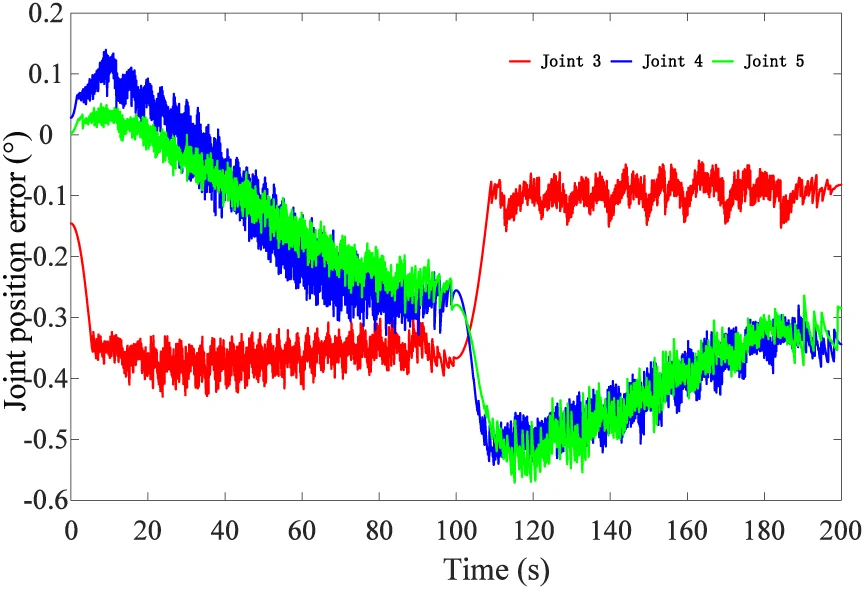
Manipulator 2 existing method joint position error.

**FIGURE 6:**
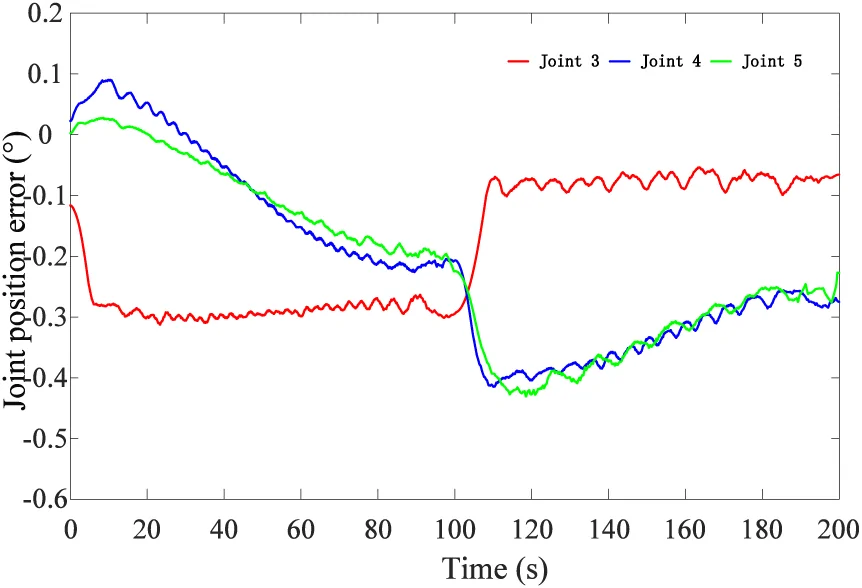
Manipulator 2 proposed method joint position error.

**FIGURE 7:**
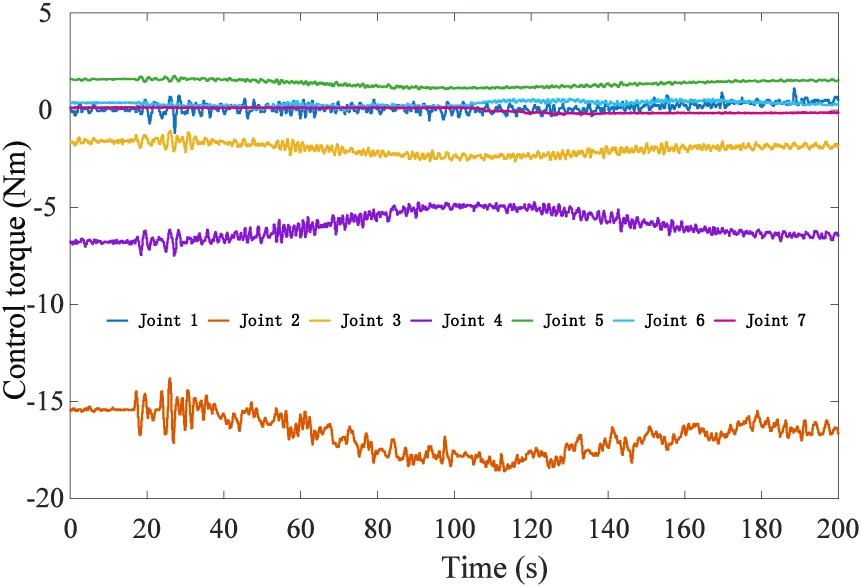
Manipulator 1 existing method control torque.

**FIGURE 8:**
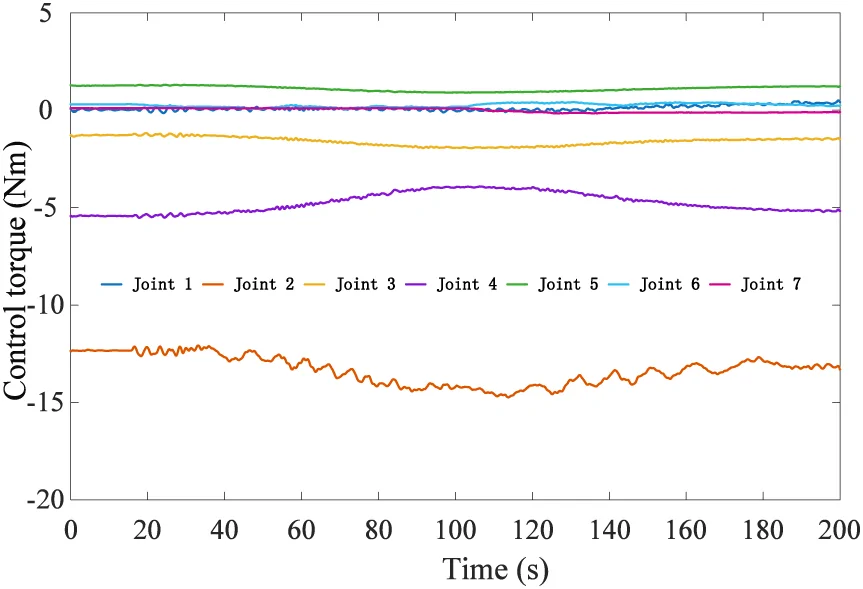
Manipulator 1 proposed method control torque.

**FIGURE 9:**
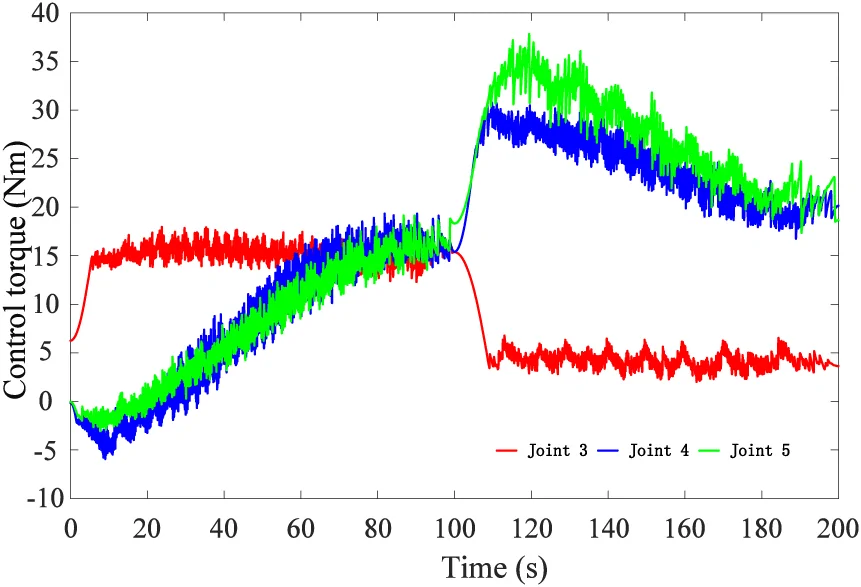
Manipulator 2 existing method control torque.

**FIGURE 10:**
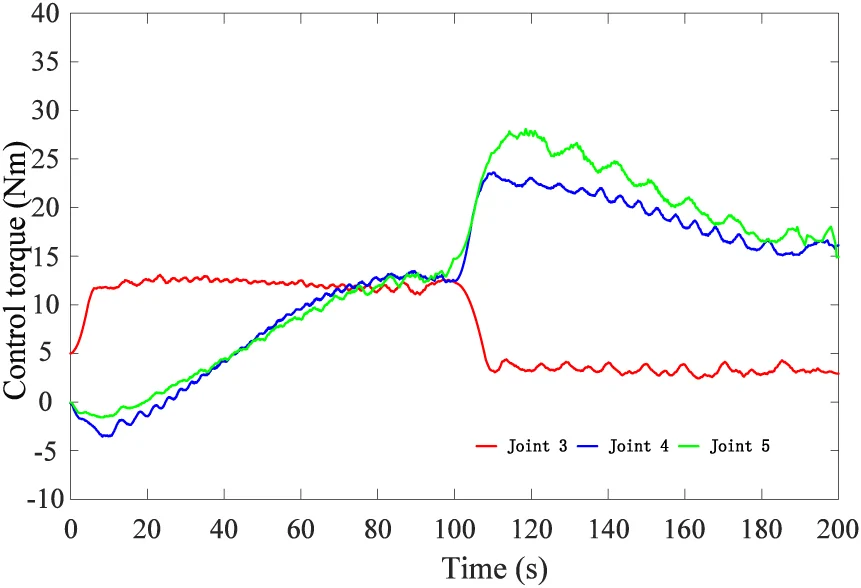
Manipulator 2 proposed method control torque.

**FIGURE 11:**
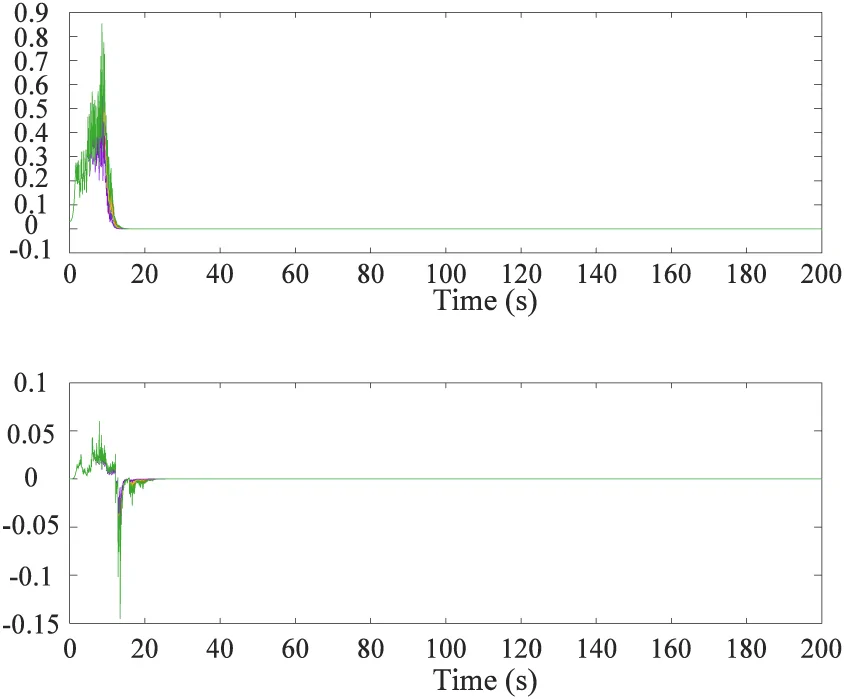
Proposed method critic NN.

Figure 2 shows the position trajectory in Cartesian space. Because the end-effector of the manipulator only moves along the Z-axis, the curve depicting the actual position of the Z-axis changing over time is presented. Figures 3–6 show position tracking error in joint space. From the figures, the desired position and the actual position are highly overlapping; hence, the position error is within a small range via the proposed method. Therefore, the effectiveness of the proposed optimal control method has been verified. Figures 7–10 show control torque curves of two manipulators by different methods. The control torque curves are smooth and gentle, and the amplitude of the control torque is within an acceptable range, ensuring good output performance of the motor. Therefore, the developed method is effective. Figure 11 is the critic NN curves. As demonstrated by the curves, owing to the learning mechanism of the neural network, the curves converge to zero within a few seconds, thereby ensuring the stability of the system.

## 5 Conclusion

This work introduces a distributed coordinated optimal control approach employing adaptive dynamic programming for dual-arm reconfigurable manipulators handling diverse objects. Kinematic and force analyses are conducted to derive the dynamics of the individual arm and the object using the joint torque feedback technique and the Newton–Euler method, respectively. A fusion error function with unknown target constraints is then formulated, which simultaneously maintains satisfactory position and internal force tracking while enabling accurate estimation of the target location. An adaptive observer is utilized to realize the distributed coordinated optimal control strategy within the adaptive dynamic programming framework. Lyapunov stability analysis demonstrates that the critic neural network weight approximation error, internal force error, and position tracking error are all uniformly ultimately bounded. Experimental results confirm the validity of the proposed methodology. The control mechanism proposed in this article is distributed control. However, for a reconfigurable robot, a decentralized control mechanism that can adapt to different configuration changes is more important. How to apply the decentralized control mechanism to the dual-arm reconfigurable robot system will be the content of our next research work.

## Data Availability

The raw data supporting the conclusions of this article will be made available by the authors, without undue reservation.
